# Incidence of hypothyroidism after treatment for breast cancer: A Korean population-based study

**DOI:** 10.1371/journal.pone.0269893

**Published:** 2022-06-16

**Authors:** Jongmoo Park, Choongrak Kim, Yongkan Ki, Wontaek Kim, Jiho Nam, Donghyun Kim, Dahl Park, Hosang Jeon, Dong Woon Kim, Ji Hyeon Joo

**Affiliations:** 1 Department of Radiation Oncology, School of Medicine, Kyungpook National University, Daegu, Korea; 2 Department of Statistics, Pusan National University, Busan, Korea; 3 Department of Radiation Oncology, Pusan National University School of Medicine, Yangsan, Korea; 4 Department of Radiation Oncology, Pusan National University Yangsan Hospital, Yangsan, Korea; 5 Department of Radiation Oncology, Pusan National University Hospital, Busan, Korea; University of Catania, ITALY

## Abstract

This Korean population-based study aimed to describe the patterns of hypothyroidism after adjuvant radiation therapy (RT) in patients with breast cancer. The Korean Health Insurance Review and Assessment Service database was searched for patients with invasive breast carcinomas. We calculated the cumulative incidence and incidence rates per 1,000 person-years of subsequent hypothyroidism and compared them using the log-rank test and the Cox proportional hazards model. Between 2007 and 2018, 117,135 women diagnosed with breast cancer with a median follow-up time of 4.6 years were identified. The 8-year incidence of hypothyroidism was 9.3% in patients treated with radiation and 8.6% in those treated without radiation (p = 0.002). The incidence rates per 1,000 person-years in the corresponding treatment groups were 6.2 and 5.7 cases, respectively. The hazard ratio (HR) in patients receiving RT was 1.081 (95% confidence interval [CI], 1.013–1.134; p = 0.002). After mastectomy, RT showed a trend toward a higher risk of hypothyroidism (HR = 1.248; 95% CI, 0.977–1.595; p = 0.076). Our study provides one of the largest population-based data analyses regarding the risk of hypothyroidism among Korean patients with breast cancer. The adjusted risk for patients treated with RT exceeded that for patients with breast cancer treated without RT. The effect was evident immediately after treatment and lasted up to approximately 9 years.

## Introduction

Breast cancer survival has steadily increased owing to screening programs and advancements in treatment. For example, in the United States, the 5-year survival rate increased from 85.5% in 1990 to 90.7% in 2013. On average, age-adjusted death rates have been falling by 1.4% each year, even after 2010 [1]. In Korea, the 5-year survival rate of breast cancer patients increased by 14% from 79.2% in 1993–1995 to 93.2% in 2013–2017 [2].

As the number of long-term cancer survivors increases and the quality of life of individuals with cancer becomes more important, the late effects of primary cancer treatment have become increasingly important. Excess cardiac and pulmonary morbidity associated with the use of radiation therapy (RT) in breast cancer has been extensively evaluated, and it has been clearly demonstrated that some of the benefits of RT diminish with respect to overall mortality [3, 4]. There is another concern regarding the potential for the development of hypothyroidism. Radiation damage to the thyroid gland can be less conspicuous, as hypothyroidism does not directly affect survival, such as in coronary artery disease or heart failure. Symptoms of hypothyroidism include fatigue, weight gain, myalgia, and depression. Untreated hypothyroidism can induce hypercholesterolemia, cognitive dysfunction, and impaired consciousness.

The European Organization for Research and Treatment of Cancer 22922/10925 and MA.20 study showed a significant reduction in mortality and recurrence of breast cancer by regional nodal irradiation in stage I–III disease [5, 6]. Based on these findings, the indication for regional nodal irradiation has expanded from patients with N2 disease to N1, resulting in an increase in the number of patients receiving RT to the supraclavicular area [7]. Studies have suggested an elevated risk of hypothyroidism in patients undergoing adjuvant breast RT, including in the supraclavicular region [8, 9]. However, only small-scale studies have been conducted, particularly in Asian women.

Therefore, the present study aimed to evaluate the patterns of hypothyroidism development among Korean patients undergoing breast cancer surgery. We sought to determine the risk of hypothyroidism in patients treated with and without radiation and the association of supraclavicular lymph node (SCL) RT with this risk. To the best of our knowledge, this is the first Korean population-based study.

## Materials and methods

### Data source

South Korea has a universal health coverage system: the National Health Insurance Service that covers approximately 98% of the country’s population. The Health Insurance Review and Assessment Service (HIRA) reviews and evaluates the appropriateness of medical expenses claimed by medical institutions. The HIRA data are the minimum necessary data for this purpose and include patient demographics (patient sex, age, and residential area) and clinical details (diagnosis, surgery, and procedures). Upon request, a dataset is provided to researchers for research purposes. The dataset is reconstructed such that it cannot be used for purposes other than those specified in the request. Each case is given an alternative code for personal identification, which is anonymized to prevent personal identification, even when linked to other data. The nationwide cohort analyzed for this study was based on the HIRA data and included patients who were diagnosed with breast cancer and underwent breast surgery between January 1, 2007 and December 31, 2018. Data on the patient’s diagnosis, age, surgery, procedure, region, and type of hospital were collected.

### Patient selection

Diagnoses were coded based on the Korean Standard Classification of Diseases, 7th revision (KCD-7). The KCD-7 code for invasive breast carcinoma is C50, hypothyroidism is E03, and subclinical hypothyroidism is E02. We excluded patients with any diagnosis of thyroid disease in the year before the cancer diagnosis.

The codes for breast surgery were identified as follows: N7121 and N7122 for excision of benign breast tumors; N7131, N7132, N7138, and N7139 for total mastectomy; and N7133, N7134, N7136, and N7137 for breast-conserving surgery (BCS). N7130 and N7135 are radical surgeries for breast cancer, including breast-conserving surgery (BCS) and mastectomy. Patients who underwent two or more breast cancer surgeries during the study period were excluded, because it was impossible to distinguish between simple reoperation, operation for recurrence, or operation for new breast cancer owing to the nature of our dataset. The codes for RT were HD05, HD06, and HZ271. We defined a fractionation approach based on the number of claims for delivering RT within 12 months after breast surgery, as the start and end dates were considered. Radiation delivery for more than 20 days was considered significant adjuvant RT.

### Statistical analysis

Baseline characteristics of patients who did or did not receive RT were compared using Student’s t-test and chi-square test for continuous and categorical variables, respectively. The time to events was calculated from the date of breast surgery to the first diagnosis on the claim. Prescriptions for levothyroxine were not investigated. Incidence was determined using Kaplan–Meier survival function estimates and compared using the log-rank test. Person-time incidence rates were calculated based on the number of events and the cumulative person-years. Multivariate analysis was performed using Cox proportional hazards model. We checked the proportionality assumption using the Kaplan–Meier estimator and the graph of log-log survival, resulting in parallel lines. The year of surgery (2013) and patient age (60 years) were selected as covariates for the multivariate analysis based on clinical judgment. The year 2013 represents the time intensity-modulated RT (IMRT) became popular in Korean breast cancer treatment [10]. The prevalence of hypothyroidism is the highest among Korean women in the 60s age group [11]. All analyses were two-sided, and a P value <0.05 was considered significant. All statistical analyses were performed using the R software (R Foundation for Statistical Computing, Vienna, Austria).

The risk of hypothyroidism in relation to radiation was assessed separately in women who underwent total mastectomy for breast surgery. The degree of radiation exposure in the thyroid gland is directly related to SCL irradiation. As the HIRA database does not provide RT field information, it is not known whether SCL RT was performed, especially in BCS cases. In contrast, in the case of RT after total mastectomy, nearly all patients were likely to receive RT in an SCL field; thus, a clearer comparison was possible. Breast surgery codes N7131, N7132, N7138, and N7139 were selected for this purpose. In addition, we categorized N7130 and N7135 codes without the related RT code within 12 months of breast surgery as total mastectomies.

This study was approved by the Institutional Review Board (IRB No 05-2020-268). The requirement for informed consent was waived by the IRB.

## Results

**Fig 1:**
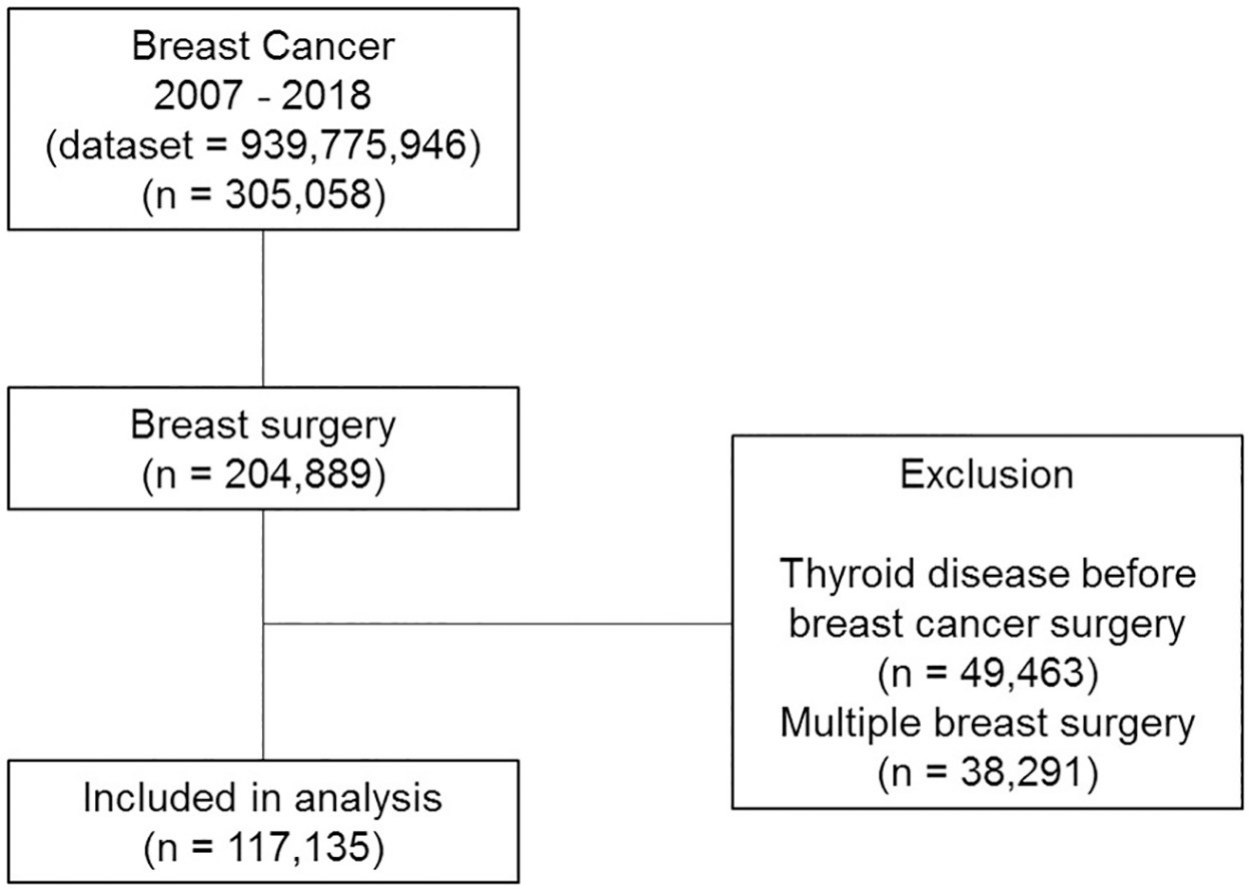
Process for inclusion and exclusion of patients in the study.

Between 2007 and 2018, the HIRA database incorporated data from 939,775,946 claims from 305,058 patients diagnosed with breast cancer. Among them, 204,889 patients underwent breast surgery. Those with any thyroid disease diagnosis before surgery (n = 49,463) or multiple breast surgery codes (n = 38,291) were excluded, and 117,135 patients with breast cancer fulfilled the inclusion criteria (Fig 1).

The median follow-up time was 4.6 years (interquartile range, 2.1–7.6 years). Patient characteristics are listed in Table 1 for patients who received RT (n = 64,080) and those who did not (n = 53,055). The mean age at surgery was higher among patients not receiving RT (52.1 years) than among those receiving RT (50.5 years).

**Table 1:** Characteristics of all patients who received RT and those who did not receive RT.

		Treated without RT	Treated with RT	*p* ^†^
		(N = 53,055)	(N = 64,080)	
Year of surgery	2008	5465 (10.3%)	4546 (7.1%)	<0.001
	2009	5492 (10.4%)	4042 (6.3%)	
	2010	5853 (11.0%)	4288 (6.7%)	
	2011	4773 (9.0%)	5639 (8.8%)	
	2012	3609 (6.8%)	6376 (10%)	
	2013	3480 (6.6%)	6669 (10.4%)	
	2014	3711 (7.0%)	6870 (10.7%)	
	2015	3917 (7.4%)	6695 (10.4%)	
	2016	4510 (8.5%)	7378 (11.5%)	
	2017	5004 (9.4%)	6932 (10.8%)	
	2018	7241 (13.6%)	4645 (7.2%)	
Age, mean, y		52.1 ± 11.5	50.5 ±10.1	<0.001
Age	≤29	686 (1.3%)	775 (1.2%)	<0.001
	30–39	5723 (10.8%)	7319 (11.4%)	
	40–49	17932 (33.8%)	23836 (37.2%)	
	50–59	15234 (28.7%)	20040 (31.3%)	
	60–69	8328 (15.7%)	9304 (14.5%)	
	≥70	5152 (9.7%)	2806 (4.4%)	
Region	Seoul	28065 (52.9%)	33101 (51.7%)	<0.001
	Busan	3576 (6.7%)	4473 (7%)	
	Incheon	1660 (3.1%)	3035 (4.7%)	
	Daegu	2670 (5.0%)	3926 (6.1%)	
	Gwangju	520 (1.0%)	365 (0.6%)	
	Daejeon	1296 (2.4%)	1317 (2.1%)	
	Ulsan	840 (1.6%)	721 (1.1%)	
	Gyeonggi	9365 (17.7%)	10071 (15.7%)	
	Gangwon	604 (1.1%)	963 (1.5%)	
	Chungbuk	365 (0.7%)	408 (0.6%)	
	Chungnam	518 (1.0%)	938 (1.5%)	
	Jeonbuk	761 (1.4%)	1451 (2.3%)	
	Jeonnam	1515 (2.9%)	1556 (2.4%)	
	Kyungbuk	178 (0.3%)	106 (0.2%)	
	Gyeongnam	965 (1.8%)	1382 (2.2%)	
	Jeju	157 (0.3%)	267 (0.4%)	
Type of hospital	Tertiary General Hospital	36347 (68.5%)	47041 (73.4%)	<0.001
	General Hospital	14700 (27.7%)	15381 (24%)	
	Hospital	1323 (2.5%)	862 (1.3%)	
	Clinic	683 (1.3%)	796 (1.2%)	
	Etc.	2 (0.0%)	0 (0%)	

*Abbreviations* RT, radiation therapy

^†^ P-value for differences between the proportion of patients treated with RT and without RT according to the treated year, age, region, and type of hospital.

### Incidence and predictors of hypothyroidism

In all patients with breast cancer, the 1-, 5-, and 8-year incidence rates of hypothyroidism were 1.3%, 5.9%, and 9%, respectively. The incidence rate per 1,000 person-years was calculated to be 6.0 cases.

**Fig 2:**
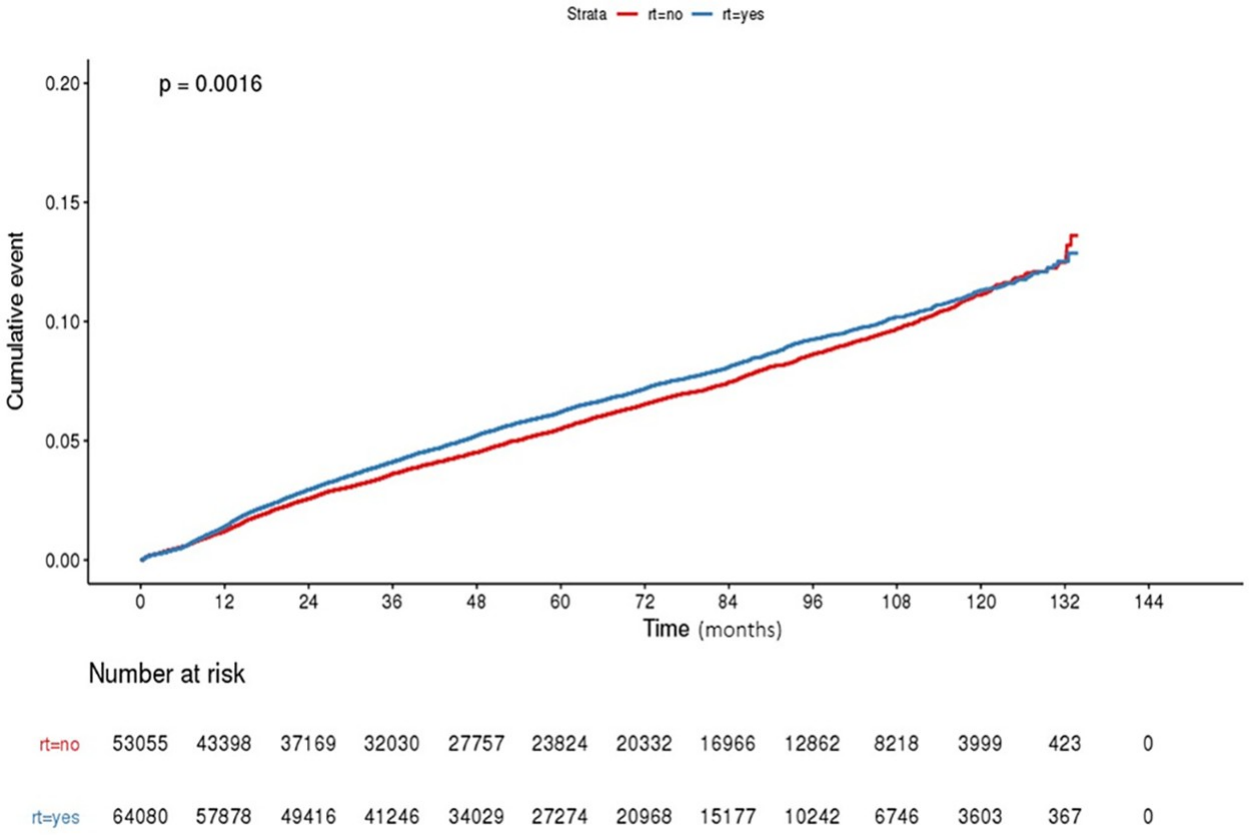
Cumulative incidence curves of hypothyroidism.

In the unadjusted analysis, the incidence of hypothyroidism differed according to the RT status (p = 0.002). For example, the 1-, 5-, and 8-year unadjusted incidence rates of hypothyroidism were 1.4%, 6.2%, and 9.3%, respectively, in patients treated with radiation. The rates were 1.2%, 5.5%, and 8.6% in those treated without radiation. The incidence rates per 1,000 person-years in the corresponding treatment groups were 6.2 and 5.7 cases. When compared to women not receiving RT, the hazard ratio (HR) for hypothyroidism in RT-treated patients was 1.081 (95% confidence interval [CI] 1.013–1.134, p = 0.002). In the cumulative incidence graph, there was a difference in the incidence of hypothyroidism immediately after treatment, and this difference was maintained for approximately 9 years (Fig 2). In multivariate analysis, after adjusting for the year of surgery and patient age as covariates, the former HR remained unaffected. Table 2 shows the results of multivariate analysis.

**Table 2:** Incidence rates, unadjusted and adjusted risk of hypothyroidism associated with RT.

Model	No RT	RT	HR (95% CI)	*p*
	Events/person-years, IR	Events/person-years, IR		
All patients				
RT	2933/511380, IR: 5.7	3782/614087, IR: 6.2	1.081 (1.013–1.134)	0.002
RT (adjusted)				
+surgery before 2013			1.097 (1.044–1.151)	<0.001
+ surgery before 2013			1.085 (1.033–1.139)	0.001
+ age under 60				
Patients treated mastectomy				
RT	1686/344550.5, IR: 5.4	67/18838.5, IR: 3.6	1.248 (0.977–1.595)	0.076
Any RT (adjusted)				
+surgery before 2013			1.293 (1.011–1.652)	0.040
+ surgery before 2013			1.267 (0.991–1.620)	0.059
+ age under 60				

*Abbreviations* RT, radiation therapy; IR, incidence rate; HR, hazard ratio; 95% CI, 95% confidence interval; HR, hazard ratio; 95% CI, 95% confidence interval; RT, radiation therapy.

A subgroup analysis was performed to assess the risk of hypothyroidism in relation to SCL RT in patients who underwent mastectomy. Among 1,936 women who underwent total mastectomy and RT, the incidence rates of hypothyroidism at 1, 5, and 8 years were 1.7%, 6.9%, and 9.2%, respectively. The rates were 1.2%, 5.2%, and 8.3% in those treated without radiation, respectively. In patients who underwent mastectomy, radiation treatment showed a trend toward a higher risk of hypothyroidism. The HR was 1.248 (95% CI 0.977–1.595, p = 0.076). This association remained marginally significant in the adjusted analysis (Table 2).

## Discussion

This study, derived from the HIRA database of the National Health Insurance Service in Korea, evaluated the incidence of hypothyroidism in patients who underwent adjuvant RT between 2007 and 2018. The incidence of hypothyroidism was higher in patients who received RT (HR = 1.081, 95% CI 1.013–1.134, p = 0.002).

In other countries, nationwide registry-based studies have been conducted to evaluate the risk of hypothyroidism in breast cancer survivors. The most recent publication was a Danish study, which included 44,574 breast cancer survivors and 203,306 matched controls. The risk of hypothyroidism was higher in breast cancer survivors than in controls (5-year cumulative incidence: 1.8% vs. 1.6%). Among the cancer treatments, RT to regional lymph nodes and chemotherapy showed the highest risks, with an HR of 1.74 (95% CI 1.50–2.02). In analyses restricted to the breast cancer cohort alone, nodal RT with or without chemotherapy was associated with an elevated risk of hypothyroidism compared to not receiving these therapies [12]. In a Canadian database study, changes in comorbidities before and after treatment were investigated in patients who received breast cancer treatment between 2005 and 2009, and this was compared with the matched cohort. The development of new comorbidities, such as ischemic heart disease, heart failure, depression, diabetes, osteoporosis, and hypothyroidism, was higher in women with breast cancer. The HR for hypothyroidism was 1.17 (95% CI 1.09–1.26) [13]. The long-term consequences of cancer treatment were assessed using a long-term British cancer survivor dataset. A total of 26,213 adults who survived for 5 years following breast, colorectal, or prostate cancer were matched with non-cancer controls. Breast cancer survivors had a marginally increased rate of hypothyroidism in multivariate models (HR, 1.26; 95% CI 1.02–1.56) [14]. Smith et al. specifically assessed this subject in the older population aged > 65 years from the Surveillance, Epidemiology, and End Results (SEER)Medicare cohort. The study included 38,255 patients with stage 0–3 breast cancer and 111,944 controls. The 1- and 5-year incidence rates of hypothyroidism were 4% and 14%, respectively. They compared the incidence of hypothyroidism among irradiated patients with 4+ positive lymph nodes (surrogate for supraclavicular RT) and no positive nodes (surrogate for no supraclavicular RT), non-irradiated patients, and controls. All patients, regardless of RT status, were more likely to be diagnosed with hypothyroidism than were cancer-free controls (hazard ratio [HR] = 1.21; 95% CI 1.17–1.25). However, supraclavicular irradiation did not appear to amplify risks, as the incidence of hypothyroidism did not increase in irradiated patients with 4+ LN versus 0 + LN (HR = 1.04; 95% CI 0.89–1.23) [9].

Our study provides one of the largest population-based data analyses regarding the long-term risk of hypothyroidism among Korean breast cancer patients, with an 8-year incidence in this patient population as high as 9%. Importantly, the adjusted risk for patients treated with RT exceeded that for those with breast cancer treated without RT (HR = 1.085, 95% CI 1.033–1.139, p = 0.001). In the subgroup analysis of patients who underwent mastectomy, the adjusted HR was 1.267 (95% CI, 0.991–1.620; p = 0.059). We confirmed that the cumulative incidence and relative effect estimates in Korean patients with breast cancer were similar to those observed in previous studies [9, 12–14].

The pathophysiology of hypothyroidism associated with breast cancer treatment has not yet been clearly established. Direct thyroid cell damage from radiation, as well as injury to small thyroid vessels and to the gland capsule, can cause RT-induced thyroid dysfunction. It has been suggested that late injury is mainly caused by vascular damage, whereas acute effects result from parenchymal cell damage [15]. There is also an explanation for the immune-mediated damage [16]. However, the contributions of other treatment modalities remain unclear. A few studies have evaluated the synergistic effects of chemotherapy, with conflicting results [12, 17, 18]. The possibility of an earlier diagnosis owing to frequent regular contact with healthcare services or symptoms of hormone therapies cannot be eliminated. As most clinical data are reported on populations treated for Hodgkin disease and head and neck cancer, the National Comprehensive Cancer Network guidelines recommend routine screening of thyroid-stimulating hormone levels, at least annually in these patients [19, 20]. In breast cancer, however, no guidelines exist on post-treatment thyroid function tests, including proper screening time [21]. Evidence indicates that antimicrosomal antibodies are elevated precociously after the end of local RT, and thyroid damage initially manifested within 6 months [16, 22]. Most reported that the events occurred within 5 years, with a median clinical latency of 8–27 months [23–27]. In our study, the effect of RT on cumulative incidence was evident immediately after treatment, and differences were observed for up to approximately 9 years. Notably, the incidence curve pattern was analogous to that of the Danish trial [12]. As symptoms are very nonspecific and are easily marked in cancer populations, it would be helpful to consider the possibility of RT-induced hypothyroidism in symptomatic patients and to consider laboratory tests for at least 5–8 years.

Intensity-modulated RT, as the most modern planning technique, usually minimizes incidental exposure to nontarget tissues and organs. However, IMRT may cause increased low-dose exposure to the thyroid gland, compared with 3D-CRT [28, 29]. The use of regional nodal irradiation, including IMRT, is expanding rapidly. Further experimental and clinical studies are required to determine the appropriate dose constraints for the thyroid gland.

In the general population, the prevalence of hypothyroidism increases with age [30]. In contrast, in the case of thyroid disorders after radiation with or without chemotherapy, susceptibility has been reported to be higher in younger patients [8, 31]. One possible hypothesis is that the upper limit of the SCL field is higher among younger women. Second, intense chemotherapy is often administered more frequently in younger patients.

Limitations of the current study include misclassification of the outcome of hypothyroidism, as it was based on diagnostic codes and did not reflect TSH elevation or levothyroxine prescription. Second, owing to data characteristics, analysis relating to the radiation dose or field, which is critical in radiation toxicity, was not performed. Third, it is possible that some patients with unclear mastectomy types were completely omitted from the subgroup analysis. Fourth, it is possible that hypothyroidism, which occurred during the interval between surgery and RT, was misclassified as an RT-induced disease, leading to immortal time bias. Fifth, some of the included patients might have undergone thyroid surgery owing to increased healthcare interactions, leading to the diagnosis of thyroid nodules or other thyroid diseases.

## Conclusion

In conclusion, the use of RT in patients with breast cancer was associated with an increased risk of hypothyroidism, which lasted for several years. The SCL-RT-confined subgroup analysis revealed similar results.
